# Web-Based and Telephone Surveys to Assess Public Perception Toward the National Health Insurance in Taiwan: A Comparison of Cost and Results

**DOI:** 10.2196/ijmr.4090

**Published:** 2015-04-17

**Authors:** Ming-Chin Yang, Elise Chia-Hui Tan

**Affiliations:** ^1^Institute of Health Policy and ManagementCollege of Public HealthNational Taiwan UniversityTaipei CityTaiwan

**Keywords:** Web-based survey, telephone survey, public perception, National Health Insurance, self-reported health status

## Abstract

**Background:**

Numerous studies have examined the impact of global budget payment systems of health insurance on patient access to medical care. In order to monitor the population’s accessibility to health services, a series of survey are often used to understand public perceptions of the health care provider. Taiwan implemented the single-payer National Health Insurance in 1995 and has been conducting a series of surveys to monitor public perception of the NHI after adopting a global budget payment system in 2002. Although telephone surveys are commonly used in obtaining public opinions on various public health issues, limitations such as higher cost and influence of interviewers do raise some concerns. Web-based surveys, one of the alternative methods, may be free from these problems.

**Objective:**

Our aim was to examine the difference of sociodemographic characteristics, satisfaction of NHI and NHI-contracted health care providers, attitude toward NHI-related issues, behavior in seeking medical advice and self-reported health status between those who completed Web-based surveys and those reached by telephone.

**Methods:**

This study compared the demographic factors of participants who took either a Web-based survey (1313 participants) or random digit dialing telephone survey (2411 participants) that contained identical questions.

**Results:**

Compared to telephone survey respondents, Web-based respondents tended to be younger (*P*<.001), unmarried (*P*<.001), non-smokers (*P*<.001), with a higher education (*P*<.001), and a higher monthly household income (*P*<.001) and worse self-reported health status (*P*=.008); however, they were less likely to report suffering from a chronic disease (*P*<.001). Despite these differences in background characteristics, no significant differences were observed in their answers related to the seeking of medical care or NHI-related issues. Telephone survey respondents reported greater satisfaction with NHI services. Web-based surveys were also shown to provide a lower average cost per sample (US$0.71) compared to telephone surveys (US$3.98).

**Conclusions:**

Web-based surveys provide a low-cost alternative method for the polling of public attitudes toward NHI-related issues. Despite general similarities between the two polling methods with regard to responses, respondents to telephone surveys reported a stronger agreement with regard to satisfaction with NHI services and a more positive self-reported health status.

## Introduction

### Background

Health care systems often use the reform of payment system to create incentives for health care providers to provide only necessary service within a limited budget which could have influence on population’s accessibility to health care. Numerous studies have examined the impact of global budget system on access to health care. Iglehart et al found that after introducing a global budget, changed allocation of local physicians affected accessibility to medical care [1]. Chu et al also indicated that active suppression of medical expenditures curtailed access to medical care due to bed closures [2]. Many countries use surveys as a monitoring measure aimed at ensuring equity in access to medical care. The Agency for Healthcare Research and Quality (AHRQ) in the United States developed the Consumer Assessment of Healthcare Provider and Systems (CHAPS) with the aim of measuring, reporting on, and improving the quality of health care from the perspective of consumers and patients [3]. Similarly, the Department of Health in the United Kingdom (UK) has been conducting surveys regarding public perceptions of the National Health Service (NHS) and the GP Patient Survey [4,5].

Taiwan implemented a National Health Insurance (NHI) system in 1995 and implemented a global budget payment system in 2002 to contain skyrocketing medical expenditures. The NHI system is a single-payer system which covers more than 99% of residents and contracts with more than 90% of the hospitals and clinics. Like other countries, Taiwan’s National Health Insurance Administration (NHIA) has been conducting a series of surveys on public perception toward the NHI since 2002. The aim of this series of surveys is to understand public perception regarding NHI and health care services and to track how perceptions have changed over time. Questions in this series of surveys comprise core and optional items. Core items are included in every iteration of the annual survey, while optional items are designed specifically for current topics of interest. Every year, telephone survey data are collected using a computer-assisted telephone interviewing (CATI) system, in which samples are selected using a random digit dialing (RDD) system.

A number of problems have been identified in these telephone surveys [6]. Evidence has shown that the gender and race of the interviewer can influence the results [7,8]. The attitude and demeanor of the interviewers may also bias the responses [9,10]. Despite the high response rates, telephone surveys are becoming increasingly expensive, which may be due to the time required to complete surveys using these methods [11]. One alternative to telephone surveys is to use the World Wide Web. Web-based surveys are more likely to be free from interviewer bias due to the nature of being self-administered. They also create a data input file directly. Information obtained from Web-based surveys displays higher test-retest reliability than do telephone surveys [12]. Previous studies have suggested that respondents to Web-based surveys are more likely to describe their personal habits such as drinking [13,14], as well as their concerns regarding traffic safety [15]. This approach also raises concern about the comparability of information obtained using Web-based surveys with that obtained by telephone surveys. Telephone surveys generally obtain a representative national sample; however, Web-based surveys are restricted to respondents who have Internet access.

### Study Aims

To the best of our knowledge, no previous study has examined the differences between Web-based surveys and telephone surveys regarding perceptions of NHI or NHI-contracted health care providers and self-reported health status. Our aim was to examine the difference of sociodemographic characteristics, satisfaction of NHI and NHI-contracted health care providers, attitude toward NHI-related issues, behavior in seeking medical advice and self-reported health status between those who completed Web-based surveys and those reached by telephone.

## Methods

### Development of Questionnaires

To obtain the data necessary to answer the study questions, we formulated a cross-sectional data collection framework using the telephone and internet as communication media. Both survey methods were conducted in August-September 2013. All people aged 20 or older in any region of Taiwan were eligible to take the survey. Screening was conducted by asking respondents whether they were 20 years of age.

The conceptual framework of questionnaires incorporated seven dimensions: satisfaction, health status, use of health service, personal health practice, need for health care, enabling resources, and predisposing characteristics. The draft version of the questionnaire was first reviewed by five experts to enhance content validity. Based on their suggestions, we modified the wording and items on the questionnaires. Then, a pre-test with small sample size was executed to examine the internal reliability of questionnaires. The Cronbach alpha coefficients of these seven dimensions were between .5 and .8. The final questionnaires included 43 items aimed at measuring satisfaction with the NHI and NHI-contracted health care providers, attitudes toward NHI-related issues, use of health service, health status (SF-8 health survey), health behaviors (eg, smoking, drinking, and chewing betel nuts), sociodemographic characteristics, and medical history.

The contents of the Web- and telephone-based questionnaires were identical in terms of the questions asked, the wording, and the order of presentation. The study protocol was approved by the ethics committee at National Taiwan University Hospital (IRB number: 1000401).

### Telephone Interview Survey

Individuals aged 20 years and older were eligible for interviews. Stratified random sampling procedure was used to draw respondents for telephone interviews. We contracted a company with experience in conducting telephone interviews; they used the RDD method with CATI to carry out the interview. All interviews were conducted from a central location, and the interviewing process was monitored by supervisors to control quality. All interviewers were well-trained individuals working on a regular basis for the survey company. The household telephone number list was obtained from Chunghwa Telecom, a major provider in Taiwan. To improve the coverage rate and increase the chances of contacting of households that were not registered in the phone book, we randomly replaced the last two digits of the drawn telephone numbers with two different digits to form a new telephone number. If there were missing items, the telephone interviewers would connect participants again to complete the questionnaires. At the end of the survey, the total number of usable responses was 2411 with a sampling error of 2.0%.

### Web-Based Survey

The sampling lists used in the Web-based and telephone surveys were obtained from different suppliers to avoid overlap. The panel list used in the Web-based survey included 320,000 individuals who were also matched to the defined target population. The respondents were randomly drawn from panel members aged 20 and older and invited via email. In addition, interested participants could click on a hyperlink to access information related to the study. The participants were required to finish all the items on the questionnaires, and then they could join the lottery. Finally, we obtained 1313 usable responses with sample error of 2.7% in the Web-based survey.

### Statistical Analysis

Data were analyzed using SAS version 9.3 for Windows (SAS Institute). Descriptive statistics were used to describe the difference between respondents in the telephone-based and Web-based groups. An independent sample *t* test and Pearson chi-square test were used to test whether the differences were statistically significant. A *P* value of less than .05 was considered statistically significant. Multivariate logistic regression and mixed linear regression models were used to test the differences in each dimension of the questionnaire by reporting their odds ratios, coefficients, and standard deviations.

## Results

The demographic and clinical characteristics of all 3724 respondents are presented in Table 1. The respondents who filled out the Web-based questionnaire were generally younger (*P*=.0001), unmarried (*P*<.001), nonsmokers (*P*<.001) with a higher education level (*P*<.001) and higher monthly household income (*P*<.001), were less likely to suffer from chronic disease (*P*<.001), and were more likely to be living in the Taipei area (*P*<.001) when compared to respondents of the telephone survey.

**Table 1:** Characteristics of respondents who participated in the Web-based and telephone surveys (n=3724).

		Telephone (n=2411) frequency (%)	Web-based (n=1313) frequency (%)
**Gender**			
	Male^a^	987 (40.9)	593 (42.5)
	Female	1424 (59.1)	720 (54.8)
**Age, years**			
	20-29^b^	231 (9.6)	410 (31.2)
	30-39	422 (17.5)	503 (38.3)
	40-49	675 (28.0)	283 (21.6)
	50-59	591 (24.5)	100 (7.6)
	≥60	488 (20.2)	17 (1.3)
	Unknown	4 (0.2)	0
**Education**			
	Junior high school and below^b^	660 (27.4)	12 (0.9)
	Senior high school	787 (32.6)	160 (12.2)
	University	837 (34.7)	840 (64.0)
	Graduated school	124 (5.1)	299 (22.8)
	Unknown	3 (0.1)	2 (0.2)
**Monthly household income**			
	No income^b^	51 (2.1)	17 (1.3)
	NT$1-29,999	336 (13.9)	126 (9.6)
	NT$30,000-$59,999	749 (31.1)	432 (32.9)
	NT$60,000-$90,000	434 (18.0)	297 (22.6)
	NT$90,000 and above	549 (22.8)	280 (21.3)
	Unknown	292 (12.1)	161 (12.3)
**Marital status**			
	Unmarried^b^	429 (17.8)	631 (48.1)
	Married or cohabiting	1688 (70.0)	598 (45.5)
	Other	287 (11.9)	73 (5.6)
	Unknown	7 (0.3)	11 (0.8)
**Religion**			
	No religion	685 (28.4)	418 (31.8)
	Buddhism	1533 (63.6)	762 (58.0)
	Christ Catholic	146 (6.1)	79 (6.0)
	Other	33 (1.4)	19 (1.5)
	Unknown	14 (0.6)	35 (2.7)
**Comorbidity**			
	Hypertension^b^	314 (13.0)	64 (4.9)
	Diabetes^b^	112 (4.7)	26 (2.0)
	Lipoid metabolism	60 (2.5)	31 (2.4)
	Heart disease^b^	64 (2.7)	11 (0.8)
**Profession**			
	Government or military employee^b^	155 (6.4)	208 (15.8)
	Company or organization employee	890 (36.9)	546 (41.6)
	Person in charge	159 (6.6)	32 (2.4)
	Licensed professional	5 (0.2)	57 (4.3)
	Professional union staff	139 (5.8)	18 (1.4)
	Farmer or fisherman	92 (3.8)	2 (0.2)
	Student	59 (2.5)	110 (8.4)
	Homemaker	471 (19.5)	85 (6.5)
	Unemployed	142 (5.9)	56 (4.3)
	Retired	285 (11.8)	31 (2.4)
	Other	0	150 (11.4)
	Unknown	14 (0.6)	18 (1.4)
**Drinking**			
	Never	845 (35.0)	447 (34.0)
	Yes	1566 (65.0)	848 (64.6)
	Unknown	0	18 (1.4)
**Smoking**			
	Never^b^	1652 (68.5)	1018 (77.5)
	Smoked, but not more than five packages	159 (6.6)	109 (8.3)
	Smoked more than five packages	600 (24.9)	175 (13.3)
	Unknown	0	11 (0.8)
**Betel nut**			
	Never^b^	1981 (82.2)	1150 (87.6)
	Tried only 1-2 times	123 (5.1)	112 (8.5)
	Eaten previously, but not in the last 6 months	202 (8.4)	41 (3.1)
	Eaten many times within the last 6 months	105 (4.4)	10 (0.8)
**Exercise in past 2 weeks**			
	No	775 (32.1)	429 (32.7)
	Yes	1636 (67.9)	844 (64.3)
	Unknown	-	40 (3.0)
**Residential area**			
	Taipei region^b^	793 (32.9)	524 (39.9)
	Northern region	352 (14.6)	201 (15.3)
	Central region	456 (18.9)	231 (17.6)
	Southern region	357 (14.8)	137 (10.4)
	Eastern region	60 (2.5)	29 (2.2)
	KaoPing region	393 (16.3)	191 (14.5)

^a^ *P*<.05.

^b^ *P*<.001.

Table 2 presents the satisfaction felt by participants toward NHI and NHI-contracted health care providers, as well as behavior patterns in use of health service. Telephone survey respondents were more likely than Web-based respondents to report a feeling of satisfaction with the NHI (58.4% vs 44.7%; adjusted odds ratio [AOR] 1.97, 95% CI 1.62-2.40) and NHI-contracted health care providers (63.4% vs 48.4%; AOR 2.39, 95% CI 1.96-2.92). No significant differences were observed between telephone and Web-based survey respondents with regard to their behavior in use of health service.

**Table 2:** Comparison of Web-based and telephone survey respondents with regard to satisfaction with NHI and patterns in use of health service.

		Telephone %	Web-based %	AOR (95% CI)^b^
**Satisfaction**				
	Satisfied with NHI^a^	58.4	44.7	1.97 (1.62-2.40)
	Satisfied with NHI-contracted health care provider^a^	63.4	48.4	2.39 (1.96-2.92)
**Use of health service**				
	Feel uncomfortable but did not seek medical care in the past 6 months (yes)	26.0	14.8	0.82 (0.67-1.00)
	The most common place to seek medical care is your city of residence (yes)	94.4	92.8	0.77 (0.53-1.14)
	Most recent case of seeking medical care using NHI card was as an outpatient visit (yes)	95.4	95.4	0.99 (0.61-1.62)
	When you need to seek medical care, you go to a clinic (yes)	14.7	14.9	0.82 (0.67-1.00)

^a^Measured on a 5-point scale from 1 (very dissatisfied/disagree) to 5 (very satisfied/agree).

^b^AOR indicates the relative likelihood that Web-based survey respondents indicated a concern for a given issue, obtained using multivariate logistic regression analysis after controlling for background characteristics (Table 1) and self-reported health status.

Table 3 illustrates the attitudes of respondents toward NHI-related issues. Results of logistic regression analysis indicate that, after accounting for sociodemographic factors, comorbidity history and self-reported health status, Web-based respondents were more likely than telephone survey respondents to be aware of the details of the NHI policy on the programs specifically designed to assist the disadvantaged (41.1% vs 25.3%; AOR 1.86, 95% CI 1.52-2.27).

**Table 3:** Comparison of respondents to Web-based and telephone surveys regarding the degree to which they were concerned with NHI-related issues.

	Telephone %	Web-based %	AOR (95% CI)^b^
Counseling patients with highest number of ambulatory visits to see doctors in a specified institution^a^	86.3	91.1	1.21 (0.86-1.70)
I am aware of the details of NHI policy aimed at assisting the disadvantaged	25.3	41.1	1.86 (1.52-2.27)
I was inconvenienced when use of health service in the past 6 months	15.6	17.5	0.75 (0.53-1.06)
Increasing the copayment for emergency visits^a^	56.8	54.6	0.92 (0.76-1.12)
Referral to another hospital under the consent of patient^a^	77.9	76.7	0.73 (0.58-0.93)

^a^Measured on a 5-point scale from 1 (very dissatisfied/disagree) to 5 (very satisfied/agree).

^b^AOR refers to the relative likelihood that Web-based survey respondents indicated a concern regarding particular issues, obtained using multivariate logistic regression analysis after controlling for the characteristics of respondents listed in Table 1 and self-reported health status.

Table 4 presents the self-reported health statuses of respondents of the two surveys. Web-based respondents generally had poorer health status with regard to physical functioning (adjusted difference: −1.73; *P*<.001), role-physical (adjusted difference: −2.25; *P*<.001), body pain (adjusted difference: −1.94; *P*<.001), social functioning (adjusted difference: −3.89; *P*<.001), role-emotional (adjusted difference: −4.06; *P*<.001) and mental health (adjusted difference: −2.94; *P*<.001). However, Web-based respondents reported a higher degree of vitality (adjusted difference: 0.94; *P*=.015) than did the telephone survey respondents.

**Table 4:** Self-reported health status of respondents to Web-based and telephone surveys.

		Telephone, mean (SD)	Web-based, mean (SD)	Unadjusted difference, coefficient (*P*)	Adjusted difference^a^, coefficient (*P*)
**Physical health**					
	Physical functioning	51.38 (6.42)	50.29 (5.12)	−1.09^d^	−1.73^d^
	Role-physical	51.22 (6.25)	49.58 (4.86)	−1.64^d^	−2.25^d^
	Body pain	56.38 (5.88)	54.95 (4.21)	−1.43^d^	−1.94^d^
	General health	45.91 (8.09)	46.62 (7.41)	0.71^d^	−0.18
**Mental Health**					
	Vitality	45.67 (8.52)	47.01 (8.21)	1.34 ^c^	0.94 ^b^
	Social functioning	52.48 (6.27)	48.16 (6.43)	−4.32^d^	−3.89^d^
	Role-emotional	49.22 (6.35)	44.84 (6.12)	−4.38^d^	−4.06^d^
	Mental health	52.44 (7.44)	49.00 (6.87)	−3.44^d^	−2.94^d^

^a^Adjusted differences refer to the adjusted mean of Web-based surveys minus the adjusted mean of telephone surveys, which was obtained using mixed regression analysis after controlling for background characteristics of respondents (Table 1).

^b^ *P*<.05.

^c^ *P*<.01.

^d^ *P*<.001.

## Discussion

### Principal Findings

This study sought to determine whether a Web-based survey could be used as an alternative to RDD telephone surveys. Our results indicate substantial demographic and socioeconomic differences between the individuals who participated in the two surveys. Our findings are consistent with previous studies, in which Web-based survey respondents are generally younger, healthier, and more highly educated than respondents to telephone surveys [16,17]. This was not unexpected, due to the fact that elderly persons still lag behind other age groups with regard to internet usage [18]. The bias did not disappear after weighting according to sociodemographic factors. This raises concerns about the accuracy of Web-based surveys and how representative the information gained is of a population.

### Comparison to Prior Work

This is the first study to compare Web-based and RDD telephone surveys with regard to different dimensions. Previous studies have demonstrated that the answers provided by respondents to Web-based surveys often differ from those provided by respondents to telephone surveys [15,19]; however, other studies showed no significant differences. Zuidgeest et al investigated differences between postal surveys and mixed-model surveys (Internet questionnaires with paper follow-up). Their findings failed to indicate any differences in global ratings of health care between respondents to the two surveys [20]. Braunsberger et al compared telephone and Web-based surveys with regard to test-retest reliability in which participants filled out two-wave questionnaires. Their results indicate that the reliability of data from Web panels exceeds that obtained from telephone survey panels [21]. Our results show that despite fundamental differences between those who took the two surveys, behaviors with regard to use of health service and attitudes toward NHI-related issues were similar, except for the awareness of assisting the disadvantaged policy in NHI. This implies that the two data collection modes are able to obtain unbiased data related to these issues. However, if one would like to assess the public perceptions of health care system and population health status, the results from different survey modes could not be compared directly.

Previous studies showed that many of the satisfaction polls done in other countries focused more on assessing consumer and patient satisfaction with the use of health services but rarely assessed the public perception on the health care system. In terms of survey modes, most of the satisfaction surveys collected data via telephone, mail, face-to-face, or mixed methods. Recently, Web-based surveys with advantages of lower cost and more time saving have been broadly used in many fields. In the UK, for example, the GP patient survey used three different methods to collect data: paper, telephone, and Web. Their results indicated that the proportion of patients using Web-based survey was increasing.

Compared to previously mentioned polls done in other countries, this study demonstrated the feasibility of combining satisfaction toward use of health service with awareness and attitudes toward selected policies. Furthermore, we used both telephone and Web-based surveys to collect responses to the same questionnaire and examined whether the responses were comparable. The results indicate that, after adjusting for respondents’ socioeconomic characteristics, use of health service, personal health practice, and health status, the satisfaction toward various issues was significantly different between respondents from telephone surveys and Web-based surveys. Moreover, results of this study also showed that respondents to the telephone survey were more satisfied with NHI and NHI-contracted health care providers than those who took the Web-based survey. Previous studies have indicated that Web-based respondents tend to give more neutral or negative attitudinal evaluations [22]. Evidence also indicates that the techniques, gender, and race of the interviewer can bias the results [7,8]. Thus, it may be reasonable to expect that individuals engaged in a direct conversation with a telephone interviewer may show more positive attitude toward the issues discussed in the survey. Our findings also indicate that Web-based survey respondents are more aware of NHI policies. This is not unexpected considering the higher level of education held by these individuals and their greater access to information via the Internet.

In terms of self-reported health status, Rivara et al investigated the model effect of health-related quality of life among parents and children aged birth to 17 years using Web-based versus telephone surveys [23]. They found that the differences caused by model effect were small but statistically significant in some of the measures. Our findings are consistent with these studies, indicating that the self-reported health status of respondents to telephone surveys was significant better than those responding to Web-based surveys, after controlling for demographic and socioeconomic characteristics, health behaviors, and comorbidity history. When matched by demographic and socioeconomic characteristics and health behaviors, the differences between the two groups remained significant.

Previous studies have indicated that the cost of and time required to complete Web-based surveys (measured per respondent) are far lower than that of other methods [20,21,24]. In this study, the data obtained from the two survey methods was collected by the same commercial research firm. The overall cost of the telephone survey included the cost of acquiring and compiling the survey data followed by preparation of a database for analysis as well as overhead and profit. The overall cost of the Web-based survey included the cost of contacting panel members, directing them to the survey site, creating a platform on which to conduct the survey as well as an allocation for overhead and profit. The average cost for each usable telephone survey sample was US$3.98, whereas the average cost of obtaining each usable Web-based survey sample was US$0.71, 17.8% of the cost of the telephone survey samples.

Nonetheless, several issues should be considered when interpreting these results. Any survey of perception and behavior is subject to various forms of self-selection and self-reported bias. As discussed earlier, the RDD telephone and Web-based surveys used in this study were not immune to these limitations. We attempted to minimize the effects of these issues by ensuring informed consent and standardized data collection procedures. Despite the fact that we employed an RDD contact method for the telephone survey, the contacted individuals were still able to choose whether to participate in the interview. Similarly, the Web-based survey, which required respondents to fill out the survey online, is prone to the issue of self-selection. Further research should address this issue by using a random assignment process to determine if the same population who agrees to be interviewed would choose a different format for the interview process.

###  Conclusions

Web-based surveys offer cost savings and efficiency in obtaining data, especially on awareness and attitudes toward NHI-related issues. But when assessing the public’s satisfaction with the health care system and population health status, survey results could not be compared directly because different methods of survey could yield different outcomes.

## Abbreviations

AOR: adjusted odds ratio
AHRQ: Agency for Healthcare Research and Quality
CATI: computer-assisted telephone interviewing
CHAPS: Consumer Assessment of Healthcare Provider and Systems
NHI: National Health Insurance
NHIA: National Health Insurance Administration
NHS: National Health System
RDD: random digit dialing
UK: United Kingdom
